# Utilizing and Valorizing Oat and Barley Straw as an Alternative Source of Lignocellulosic Fibers

**DOI:** 10.3390/ma15217826

**Published:** 2022-11-06

**Authors:** Marc Borrega, Ville Hinkka, Hanna Hörhammer, Kirsi Kataja, Eija Kenttä, Jukka A. Ketoja, Rosa Palmgren, Minna Salo, Henna Sundqvist-Andberg, Atsushi Tanaka

**Affiliations:** 1 VTT Technical Research Centre of Finland Ltd., FI-02044 Espoo, Finland; 2 Department of Chemical Engineering, Mid Sweden University, SE-85170 Sundsvall, Sweden; 3 Supply Chain Management and Social Responsibility, Hanken School of Economics, FI-00101 Helsinki, Finland

**Keywords:** straw, cellulose, fiber, material, pretreatment, bonding, foam forming, hot pressing, logistics, business

## Abstract

The transition to sustainable, biodegradable, and recyclable materials requires new sources of cellulose fibers that are already used in large volumes by forest industries. Oat and barley straws provide interesting alternatives to wood fibers in lightweight material applications because of their similar chemical composition. Here we investigate processing and material forming concepts, which would enable strong fiber network structures for various applications. The idea is to apply mild pretreatment processing that could be distributed locally so that the logistics of the raw material collection could be made efficient. The actual material production would then combine foam-forming and hot-pressing operations that allow using all fractions of fiber materials with minimal waste. We aimed to study the technical features of this type of processing on a laboratory scale. The homogeneity of the sheet samples was very much affected by whether the raw material was mechanically refined or not. Straw fibers did not form a bond spontaneously with one another after drying the sheets, but their effective bonding required a subsequent hot pressing operation. The mechanical properties of the formed materials were at a similar level as those of the conventional wood-fiber webs. In addition to the technical aspects of materials, we also discuss the business opportunities and system-level requirements of using straw as an alternative source of lignocellulosic fibers.

## 1. Introduction

In Northern Europe, forest use has been the backbone of the circular bioeconomy, contributing to sustainable development, including climate change mitigation, as bio-based materials can replace fossil-based materials [1,2]. The forests are recognized to be crucial in adopting and fighting against climate change and reaching climate neutrality, as well as protecting and restoring biodiversity. Several policies in the European Union (EU) have positioned forest use at their center of attention, including the latest European forest strategy [3], land use and forestry regulation (LULUCF), the European climate law [4], and the biodiversity strategy [5]. Competition for forest raw materials will accelerate derived to tendencies such as increased use of wood as a building material, bioenergy demand, and substitution of plastic packages with paper and board-based materials. The interest in using agricultural residues as an alternative source of cellulosic material for wood cellulose is apparent.

The circular bioeconomy has already intensified the use of agricultural residues. Currently, most biorefineries in the EU use agricultural resources and residues, apart from Finland, Sweden, and Portugal [6]. During the past five years, there has been a rise in pilots and full-scale biorefineries using straw as a feedstock. In Europe, the chemical company Clariant [7] has established a cellulosic ethanol plant in Romania, while the hygiene and health company Essity [8] began pulp production based on wheat straw in Germany in 2021. In addition, there are further projects ongoing, as Fortum [9] is developing biorefining technologies and investigating new applications, such as textiles for straw-based materials. Red Leaf Pulp [10,11] is building a pulp mill in Canada, which is anticipated to use 290,000 tons of wheat straw to produce 182,000 tons of pulp and 95,000 tons of co-product per year. Biorefineries provide economies of scale but also require a sufficient supply of agro-waste and residues, due to which the current exists in the regions for securing adequate supply. Traditionally, straw has been used for centuries in various applications, such as soil incorporation, livestock bedding, construction, insulation, and power and energy generation. New applications are actively sought after, and straw is seen to have potential as a raw material to produce, for example, waxes and biochemicals, strawboards, and garden mulches (Table 1).

This interdisciplinary paper aims to broaden the understanding of the challenges and opportunities of using barley and oat straw as an alternative source of cellulose fibers. Barley and oat are the main cereals cultivated in Finland, with an annual production of about 1.4 and 1.2 million tons in 2020. They are also among the most cultivated cereals in the world, with a global production of 157 and 25.2 million tons [29]. The study focuses particularly on geographic areas, such as Finland, where straw availability and long distances challenge large-scale biorefining. Thus, collecting and transporting agricultural side streams for further refining is a key point when evaluating side streams’ business potential. When considering the utilization of straw in new products, mold development might be a major challenge. It is also essential to consider raw material adequacy and uniform quality when evaluating business potential.

To overcome these challenges, we investigate a concept where a relatively mild alkaline liquid pretreatment of oat and barley straw provides fiber fractions that can be taken to subsequent material-forming processes. We have investigated how these fractions can be used to prepare strong fiber materials without further additives. In this concept, the pretreatment processes can be distributed in smaller local units, whereas the material forming operations could be centralized.

Both the rough, unrefined straw qualities and the finer mechanically refined fractions were studied here. The extensive size distribution of fibers require special operations such as foam forming [30] to distribute the fibers uniformly over the formed material. Such a method has been used earlier to make nonwovens or webs from natural wood fibers. Despite similar chemical composition to wood, straw fibers’ bonding properties differed from common papermaking pulps. In particular, straw fibers did not form bonds spontaneously with one another after the removal of aqueous foam and material drying. This was probably caused by the larger dimensions, smoother and less fibrillated surfaces, and different surface chemistry of straw fibers when compared to chemical or mechanical wood pulp. However, similar strength as for paperboard could be achieved with hot pressing, which has been shown to increase lignin-rich wood fibers’ dry and wet strength [31,32,33]. The most potential application opportunities and logistics requirements that are important when utilizing straw materials as an alternative source of lignocellulosic fibers are discussed based on these observations.

## 2. Materials and Methods

### 2.1. Straw Materials and Their Processing

Oat (*Avena sativa*) and barley (*Hordeum vulgare*) straw were provided by the Natural Resources Institute of Finland (LUKE) after machine harvesting and collecting from the field in Jokioinen, Finland, at the end of August 2021. Upon delivery, the straws were air-dried for 6 days and then chopped using a Weima shredder (10 mm screen).

**Figure 1:**
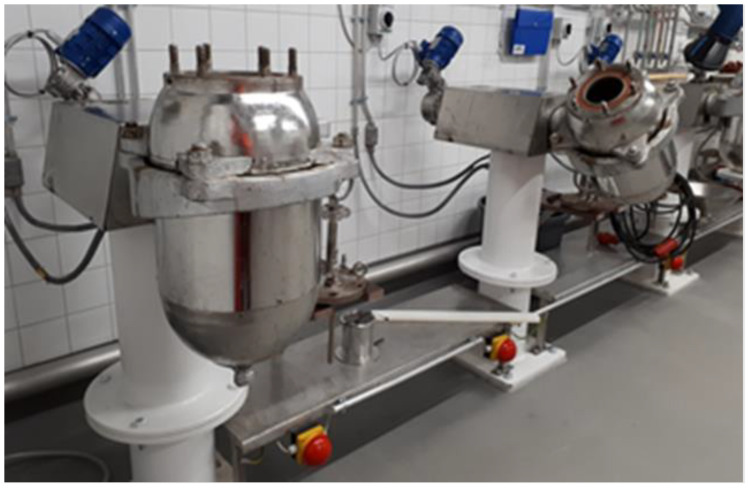
The straw material was processed in 15 L reactors.

The alkaline processing of oat and barley straw material was performed using rotating 15 L reactors (Figure 1). The experiments were conducted at two different temperatures (80 °C and 160 °C) to achieve less and more processed straw material. 470 g dry straw material was charged into the reactor, the liquor-to-straw material ratio was 6, the heat-up time 60 min, and the time at temperature 60 min. Two different sodium hydroxide (NaOH) charges were applied, i.e., 12% and 18% of the material. One experiment was performed on barley (80 °C, 12% NaOH), whereas oat was processed in the other experiments.

After the treatment, the liquid fraction was filtered, and the solid fraction was washed using cold water. The pH was measured from the liquid fraction. The process yield was determined. Half of the solid fraction was further refined using the Bauer refiner (40 mils). Kappa number (ISO 302; Metrom tiamo titrator) and brightness (ISO 2470, measured from a split sheet with no pH adjustment using L&W Elrepho spectrophotometer with L&W Colour-Brightness software Autoline^®^ 300 v 2.55.3 (AB Lorentzen & Wettre, Kista, Sweden); wavelength 457 nm, UV-level D65 for calibration and C for measurement) were determined for the solid fractions. Solid samples were analyzed for chemical composition and tested for bonding (Section 3).

### 2.2. Chemical Analyses

The extractives content in the oat and barley straw samples was determined after extraction with acetone, and the carbohydrate and the lignin content of the pre-extracted straw samples were determined after two-stage acid hydrolysis, adapted from the NREL/TP-510-42618 method issued by the US National Renewable Energy Laboratory. Neutral monosaccharides in the acidic hydrolysate were quantified by high-performance anion exchange chromatography with pulsed amperometric detection (HPAEC-PAD) in a Dionex ICS-6000 instrument (Thermo Fisher Scientific, Waltham, MA, USA) equipped with a CarboPac PA1 column. The monosaccharide content was not corrected for losses due to degradation during hydrolysis. The acid-insoluble (Klason) lignin was determined gravimetrically after the two-stage acid hydrolysis, and the acid-soluble lignin was determined in a Genesys 180 UV spectrophotometer (Thermo Fisher Scientific, Waltham, MA, USA) at 215 nm and 280 nm [34].

The ash content in the straw samples was determined gravimetrically in a Nabertherm N-11 oven after the combustion of the samples at 550 °C for 23 h. The elemental composition (C, H, N, S) was determined with a FLASH 2000 series organic elemental analyzer (Thermo Fisher Scientific, Waltham, MA, USA); more details on the analytics can be found in Ref. [35]. The protein content was quantified as the nitrogen content multiplied by 6.25.

### 2.3. Preparation of Straw-Based Fibre Materials

**Figure 2:**
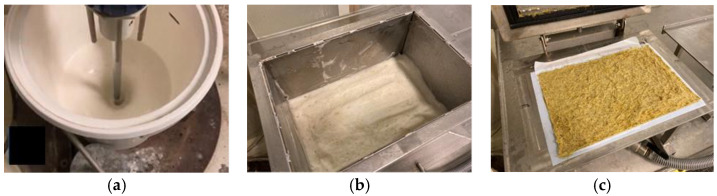
Laboratory sheet making: (**a**) Stirring of wet fiber foam with a foaming impeller; (**b**) foam poured in the mold; (**c**) wet sheet formed on a membrane filter after drainage. The sheet dimensions were 385 mm × 265 mm.

The straw fibers are quite long, and to avoid their flocking, we produced laboratory sheets with the foam-forming procedure [13]. In this method, aqueous foam is used as a medium to transfer fibers into a mold (Figure 2). Foam bubbles keep fibers apart until they deposit on a homogenous planar network. First, the total solids of 12 g of raw materials were mixed with 3 L of water and 12 g of 10% aqueous solution of sodium dodecyl sulphate (SDS) surfactant. The mixing was performed with a special foaming impeller [36] at a rotation speed of 4400 rpm. The mixing was continued for at least 3 min until the foam volume stabilized. Once the flow vortex closed up and the foam’s top surface no longer rose, the fiber foam was decanted into the mold [13] and filtered through a membrane film (SEFAR PETEX 07-1/2, Sefar AG, Heiden, Switzerland). The applied vacuum level during foam drainage was c.a. 0.6 bar. Using the membrane instead of normal papermaking forming fabric was necessary to achieve high retention of straw fines within the formed structure. The dimensions of the obtained sheet were 385 mm × 265 mm (area 0.1 m^2^), and the grammage was 120 g/m^2^.

After the above foam-forming operation, another membrane film was put on the wet sheet, and two blotting papers further sandwiched this structure. The whole laminate was dried for 2 min between metal plates at 200 °C. The clearance between the plates was 1 mm, so some light compression was applied during drying. After drying, the membrane films were carefully peeled off from the sample.

**Figure 3:**
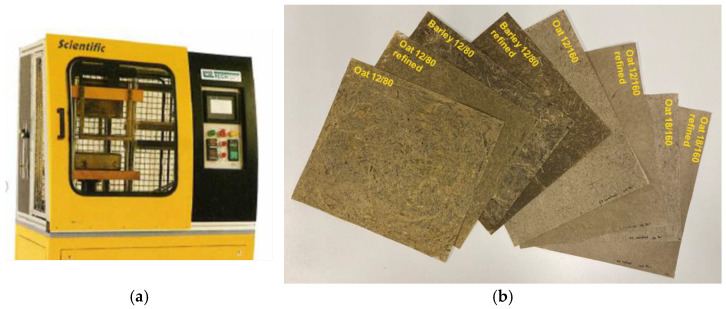
(**a**) Laboratory pressing device; (**b**) examples of produced sheets.

The strength of the foam-formed material as such was very low. In other words, similar bonding mechanisms as those found in papermaking were absent for the straw material. As the lignin content of the straw was relatively high, we decided to use heat treatment to enhance the bonding of the fibers. Hot pressing [31,32,33] has been shown to lead to high dry and wet tensile strength for lignin-containing wood pulps. In that case, the strength increase is caused by the interdiffusion mechanism [37,38] that occurs at temperatures exceeding the softening temperature of lignin (c.a. 150 °C). In our case, a dried sheet was hot pressed at 200 °C for 20 s using the pressure of 3 MPa. The blotting papers and membranes were removed before the pressing so that the sample had direct contact with heated metal plates. The hot pressing was performed with a laboratory pressing device (LP-S-50, Labtech Engineering Co. Ltd., Samutprakarn, Thailand) (Figure 3). In order to check the impact of pressing time, one of the oat sheets was pressed over an extended period of 30 min.

In principle, enzyme application could enhance the fiber bonding by increasing lignin mobility further during heat treatment. For one trial point, Trametes hirsuta laccase (ThL) was employed for this purpose. Following the study by Widsten et al. [39], 400 nkat ThL/g fiber was dosed. Since ThL has an optimal pH of 4.5, it was diluted with a 25 mM succinate buffer solution (pH 4.5). The application was made by careful spraying onto one of the Oat 12/80 sheets in a wet state (after formation). We waited for 1 h between spraying and drying.

### 2.4. Characterisation of the Formed Sheets

The surface structure of the formed sheets was imaged using a scanning electron microscope (SEM) of type ZEISS Merlin (Carl Zeiss GmbH, Jena, Germany). The FE-SEM with a secondary electron detector was operated at an accelerating voltage of 2 kV on gold-sputtered samples.

The structural and mechanical properties of straw sheets were measured according to ISO standards (Table 2). Wet strength was measured after 1 h of water immersion. 10 parallel measurements were included for each case.

The contact angle measurements were carried out with an optical Theta tensiometer (Attension, Biolin Scientific, Espoo, Finland). The unit included a camera and lenses, a light source, and a sample stage. A drop (5–6 µL) of Milli-Q water was placed onto a sheet, and the shape of the drop was followed with time intervals of 0.3 s. This measurement was carried out for both the top side and the bottom side (against the membrane film in sheet forming) of a sheet using 5 parallel measurements in each case. We report both the average immediate (0–0.9 s) and long-time (10–11 s) values of the contact angle.

The surface IR spectra of the samples were measured using Nicolet iS50 FTIR spectrometer (Thermo Fisher Scientific Inc., Waltham, MA USA) and a single reflection diamond ATR (attenuated total reflectance) crystal. The background spectrum was recorded from the clean diamond crystal. The IR spectra were collected by averaging 32 scans at a resolution of 4 cm^−1^. Three IR spectra were measured from the top side of a sample and 2–3 spectra from the bottom side.

### 2.5. Evaluation of Potential Applications and Business Cases

The main research methodology used in the evaluation of potential applications and business cases was a literature review. This is a good approach to systematically collecting previous research [40] and integrating the findings and perspectives from many empirical findings [41]. Based on the literature review (Table 1), ten potential applications for using straw cellulose fibers were identified.

The literature review was enriched with data from three semi-structured interviews and an expert workshop. Interviewees included key persons regarding the use of straw as an alternative cellulose source in Finland. The participants in the expert workshop consisted of some of the key researchers in this field in Finland. Based on the literature review, interviews, and the expert workshop, the potential applications and business cases for the use of straw as an alternative cellulose source were identified. The analysis also covered the potential strengths, weaknesses, opportunities, and threats related to straw use (Appendix A).

### 2.6. Analysis of Logistics

The data sources for the logistics analysis of straw collection consisted mainly of semi-structured qualitative interviews as well as a literature review. The role of the interviews was to give a more precise overview of the circumstances and characteristics of Finnish agriculture. The literature served as a basis for understanding the procedures and costs. Based on the collected materials, the calculations were made for the logistics of collecting the straw and their profitability.

## 3. Results

### 3.1. Chemical Composition of Straw Raw Materials

The conditions and results from the straw processing experiments are presented in Table 3. Oat and barley straw behaved similarly during processing. Processing at 80 °C resulted in yields of 5% and kappa numbers of 49–50. Naturally, the process yields and kappa numbers were lower after processing at a higher temperature of 160 °C, 58% versus 46% (yield) and 49% versus 17% (kappa number). Increased alkali charge (from 12% to 18% of the material) resulted in a lower kappa number, which indicates lower lignin content.

**Figure 4:**
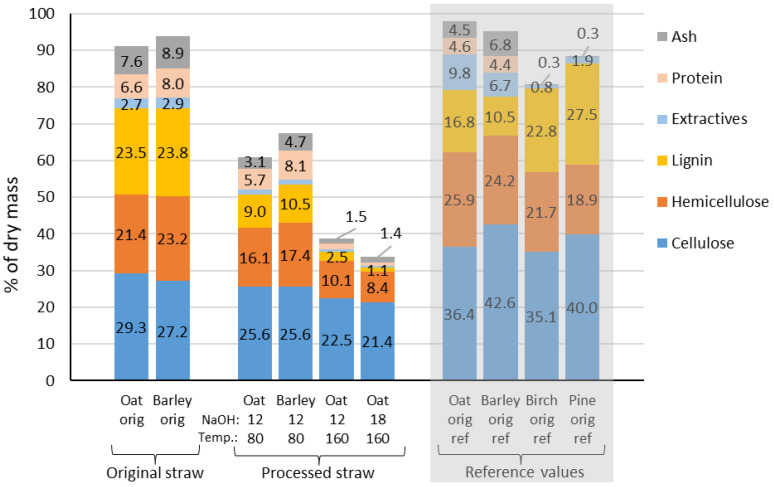
Chemical composition of oat and barley straws before and after processing. The chemical analyses were carried out for processed samples obtained from small-scale trials conducted in 1 L reactors with corresponding conditions to the 15 L trials. The processing yield has been accounted for in the processed straw’s chemical composition. Reference values (grey box) for the chemical composition of oat and barley straw are obtained from Refs. [42,43,44,45,46,47,48,49,50] and for birch and pine wood from Ref. [52].

The chemical composition of the original oat and barley straw materials is presented in Figure 4. The cellulose content was between 25–30% of the dry mass, somewhat lower than previously reported in the literature [42,43,44,45], particularly for barley. On the other hand, the lignin content was about 23% of the dry mass, much higher than reported in some studies [44,46,47], although in line with other studies [45,48,49,50]. The lignin content was likely overestimated by the presence of inorganic compounds (i.e., ash) and protein, which accounted for about 15–20% of the total dry mass. The main inorganic compound in straw is silica, which is often detrimental to straw’s chemical and enzymatic processing. Still, it may also be a valuable component for many applications [51]. In general, the chemical composition of the oat and barley straw was in relatively good agreement with the literature values, considering the natural variability of these materials and the different analytical procedures used in different studies.

The chemical composition of birch (hardwood) and pine (softwood) is presented in Figure 4 for comparison purposes. The cellulose, hemicelluloses, and lignin content of the straws were similar to that of birch wood, and the primary sugar in the hemicellulose fraction was xylose, as xylan is the predominant hemicellulose in herbaceous plants and hardwood species. Compared to pine wood, the straws contained less cellulose and lignin but had somewhat similar hemicellulose content. However, the main hemicellulose in pine and softwoods is galactoglucomannan, composed predominantly of glucose, mannose, and galactose sugar units. One of the most significant differences in composition between crop plants and tree species in the Northern hemisphere is found in the ash and protein content. Protein is considered to be a minor component of wood (<0.5%) [53].

The chemical composition of the processed material where the processing yields have been considered (Figure 4) shows that alkaline treatment removes all compounds to some extent. Processing at a temperature of 160 °C efficiently removes lignin, extractives, protein, and ash, while certain hemicelluloses and most cellulose remain in the straw material. The alkaline treatment similarly affects oat and barley material based on chemical composition. Treatment applying a higher alkali charge further increases the removal of compounds from straw material; for example, a reduction in lignin content can be observed (2.5% versus 1.1%).

### 3.2. Properties of the Formed Sheet Samples

**Figure 5:**
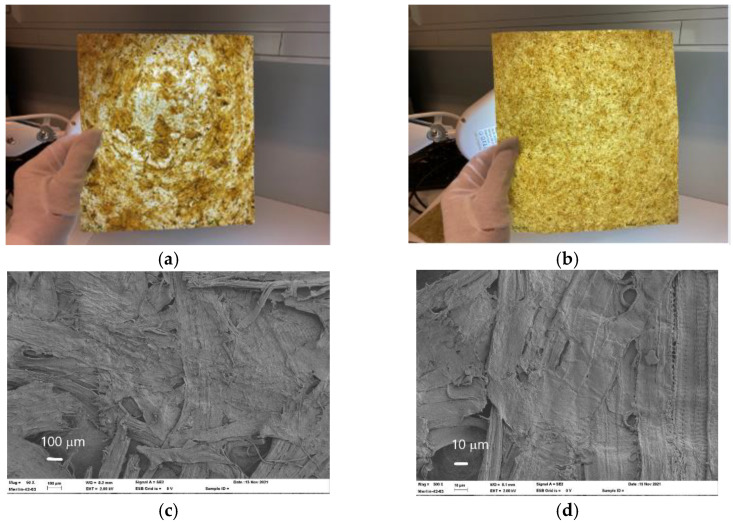
(**a**) Sample made with rough oat fibers (Oat, 80 °C, 60 min, NaOH 12) viewed against backlight; (**b**) sample made with the refined quality of the same fibers; (**c**,**d**) Hot pressing improves bonding and closes a part of the sample surface, as shown in the SEM images of the sample obtained from unrefined Oat 12/80 (Table 3).

The heterogeneous distribution of unrefined straw fibers in a formed sheet can be seen clearly in Figure 5a when viewed against backlight. In this case, the deposition of finer fiber fractions take place at sheet areas that lack large fibers. A more homogenous sheet structure was achieved by using refined fibers with a more confined size distribution (Figure 5b). The improved bonding of fibers caused by hot pressing can be seen in the microscopic images in Figure 5c,d. The densities of the samples after hot pressing varied from 500 to 670 kg/m^3^ (Table 4).

**Figure 6:**
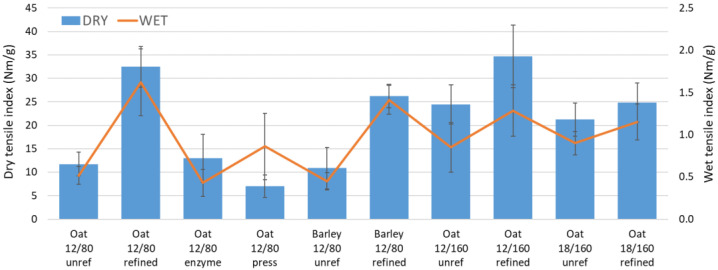
Dry and wet tensile indices of the straw sheets for varied preprocessing conditions. The hot-pressing time was 20 s for all other cases except the rightmost trial point, for which a long pressing of 30 min was applied.

The measured strength properties of the various samples are summarized in Figure 6 and Table 4. Strength was improved by refining in all cases because of a more homogeneous structure (Figure 5a,b) and improved bonding achieved with small fiber fractions. The general level of tensile index was at a similar level as for board made from typical chemithermomechanical wood pulp (CTMP) at equal density [31]. Without refining, significantly better dry strength and stretch at break were obtained using the higher 160 °C pretreatment temperature than 80 °C. In other words, the harsher chemical pretreatment partly compensated for the lower amount of small fiber fractions in the furnish. After refining, pretreatment temperature did not have a similar effect on the tensile index. Additional enzyme treatment did not significantly impact strength properties, which suggests that the applied thermal treatment alone was sufficient to cause the mobility of amorphous polymers. Interestingly, a lower level of the applied NaOH led to clearly higher dry strength for the refined oat-straw sheet than what was achieved with the higher amount.

**Figure 7:**
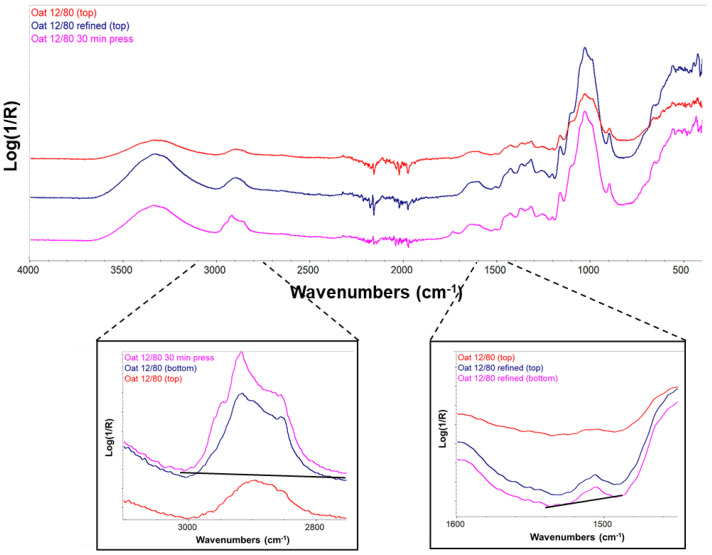
Comparison of IR spectra of different types of Oat 12/80 sheets. The IR spectrum of the top side of the unrefined Oat 12/80 sheet (red curve) shows cellulose only. The IR spectra of the bottom side of this sample and the sheet with 30-min hot pressing (left blowup) show additional weak bands of long hydrocarbon chains in the wavenumber range of 2800–3000 cm^−1^. This explains the higher values of the contact angles for the latter cases in Table 4. A small proportion of lignin is seen in the refined Oat 12/80 sheet on both sides, as the IR spectra show a small band of the aromatic ring near the wavenumber of 1510 cm^−1^ (right blowup).

The wet strength stayed at a low level despite the heat treatment. This observation was contrary to the earlier results for mechanical wood fibers [16]. The time needed for major lignin interdiffusion [37,38] may be longer for the straw raw materials, whose surface properties and dimensions differ from those of wood fibers. In addition to cellulose, the IR spectra (Figure 7) showed a weak band of the aromatic ring, characteristic of lignin [54], only in the sample made from refined oat fibers (Oat, 80 °C, 60 min, NaOH 12). This may explain the slightly higher wet strength measured for this sample. The extended 30 min hot pressing time of this sheet made its surface glossier and quite hydrophobic (Table 4), which seems to arise from the appearance of long hydrocarbon chain compounds at the sample surface as seen in the IR-spectra (Figure 7). These IR bands typically originate from waxes and oils. However, wet strength did not improve similarly as for wood fibers despite fiber hornification seen as higher solids content after immersion in water (Table 4). Moreover, dry strength dropped from the level obtained with shorter pressing. This suggests that significant amounts of hemicelluloses degrade with extended heat treatment [37].

Curiously, a similar long-chain hydrocarbon component (Figure 7) seemed to hydrophobized (contact angle 92°) and also the bottom surface of the unrefined Oat 12/80 sample (Table 4). However, such a component was not found at the top surface, for which the contact angle was clearly smaller (74°).

## 4. Discussion

### 4.1. Potential Applications and Business Cases

The increasing pressure for using residue straw comes from the policy drivers emphasizing environmental sustainability, the economic aspects favoring resource efficiency, and the need to find alternative raw material sources for wood cellulose. Several producers particularly emphasize the environmental benefits of straw, as it is a by-product of cereal production. Thus, this residue raw material is suggested to reduce the carbon footprint compared to wood pulp production with low impacts on land use or biodiversity [8,10,55]. Moreover, the use of a residual straw can improve material efficiency [55], support local economies [56], and green growth in general.

Despite the increasing business interest in straw utilization, several uncertainties still exist that can affect business opportunities. The challenges related to the use of straw entail material supply-related factors, such as limited straw availability and supply security, and ensuring sustainable harvesting [56]. There are some challenges also related to supply chain management, including the lack of infrastructure for collection and storage, as well as challenges in logistics [57]. In addition to these, economic factors, such as low business maturity and insufficient economic incentives, also challenge the use of straw as an alternative cellulose source. Table A1 (Appendix A) summarizes the potential strengths, weaknesses, opportunities, and threats related to using straw as an alternative cellulose source.

Currently, the range of straw-based product applications is vast. It ranges from using straw in its natural form to value-added products, such as chemicals or textile fibers, to large-scale biorefining, as Table 1 shows. The chemical and physical properties of straw material and the bonding tests (Section 3) indicate that mild pretreatments might be sufficient to enhance the properties of straw so that it can be used in specific applications that do not require intensive biorefining. Based on these results, a review of current applications (Table 1), and the volume of replaced wood fibers in the application, we identified ten potential applications for using straw cellulose fibers. The identified potential applications are wipes, anti-algae products, fiber-based packages, nonwoven mulches, clay-fiber composites in construction, growth medium, insulation material, and enzymatically decomposed intermediate products for targeted applications, and textile fibers.

Insulation material and takeaway packaging appear as two of the most attractive applications because they are foreseen to have steady market growth. Despite the consumption reduction measures, the global market for on-the-go single-use food packaging is projected to have over a 6% compound annual growth rate (CAGR) within the next 10 years [58]. The demand for building insulation materials is foreseen to have a CAGR of 4.5% between 2016–2027. The main drivers for the demand are greenhouse gas emission reductions, cost efficiency, and government regulations on energy-efficient buildings [2]. However, for such applications, the material should be formed without hot pressing to avoid densification that deteriorates the insulation properties.

Identifying relevant applications and market demand is essential when building a business case using residue straw. However, developing a business model requires considering market demand and other key elements, such as raw material supply, storage, logistics, and processing. The entire value chain for using residue straw as an alternative cellulose source should be considered when building a business case. All the actors involved in the value chain should be motivated. The division of work and the responsibilities and revenues between the actors should be clear to make the business case successful. As the residual cereal straw raw material base in Finland is limited, the most viable case could be smaller-scale distributed production, potentially with a temporary on-site pretreatment system and further collection of raw materials for final processing.

### 4.2. Logistics

The value chain for using straw as a raw material for cellulose starts by collecting the straw from the field. Usually, the straw is left on the field with a longer length. However, when the straw is collected for further use, it is cut lower and is not chopped. Therefore, the farmer needs to know whether the straw will be collected, as the procedure differs from the usual collection. Balers are the most efficient for straw collection, facilitating handling, and storage, primarily used at cattle farms for bailing pasture grass but usually unnecessary machinery at crop farms. The pasture grass is collected three times during the summer, remaining unused after that, and would be available to rent for crop farms for bailing straw. Another possibility is using bailing offered as a service.

The yield varies and is difficult to estimate beforehand. For a steady flow of the material, there should be flexibility in the farms involved as raw material providers. Another challenge is the quality of the material since increased rainfall during the autumn may decrease the raw material quality. Farmers aim to harvest material after a few days without rain, but this is not always an option during rainy autumn. Plastic coating during bailing facilitates sustaining the straw quality. However, the plastic coating is unnecessary for cellulose production if the bales are transported from the field soon after bailing to avoid mold formation.

The next step after collection and possible short-term storage of the bales at the edge of the field is to transport the bales to a nearby depot, preferably a central spot from the farms in the area with a maximum distance of 25 km. Based on the interviews, this is the distance where the farmers could contribute to transporting the bales from the farm to the depot by using their own machinery such as tractors, buckets, or trailers. This arrangement would be similar to the simulation model paper by Thorsell et al. [59] and the idea of an integrated biomass logistics center proposed by Suardi et al. [60]. Thus, there would be no need to drive by truck to the fields, which is especially harmful during rainy autumn, making deep tire marks on the field.

When transporting bales forward from the depot to the cellulose factory, the bale volume is a restricting factor in transport. In Finland, the most cost-effective transport alternative is to use so-called HCT (High-Capacity Transport) trucks for long-distance transport with specific prerequisites. Another option is to have local pretreatment at the depot. The pretreatment would extract material to be easier to transport over long distances. Based on the research conducted, the local processing yield would be between 41–58%, depending on, e.g., the temperature used and reagent concentration. Pretreatment will increase the density so much that weight becomes the restricting factor for transportation.

**Figure 8:**
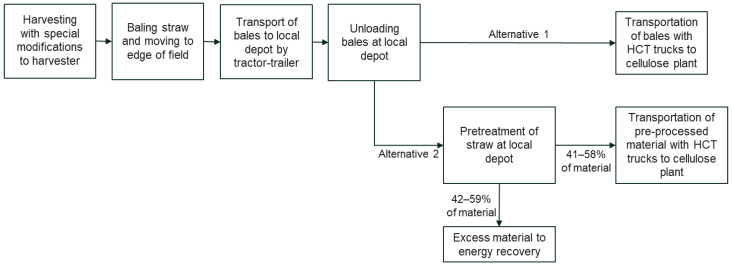
Alternative logistics depend on whether the pretreatment of straw is carried out locally or not before forming the actual material.

Then, the straw volumes and related handling costs for different alternatives (Figure 8) are estimated. In Southern Finland, 20–30% of the landscape is used for agriculture. Over half of that is used for growing grain. Therefore, when the depots are placed in local municipal centers in the countryside, it is assumed that when drawing a circle of about a 15 km radius around this depot, we can assume that at least 5% of the area around this depot is that kind of field, where it is possible to collect straws for this purpose. The harvest in Finland varies a lot annually depending on the weather conditions. Still, the average amount that a field produces in a hectare would be around 4000 kg of straw, approximately 10 bales. Farmers have trailers customarily used for transporting grain. These trailers can transport 10 bales on average. The amount of straw collected from this area is 5% × 4000 kg × πr^2^ ≈ 14,000 ton ≈ 40,000 bales.

We have used the following assumptions related to costs. The production cost of one bale and removing it to the edge of the field would be €10–11; if the plastic coating is needed, the cost will be €13–14 [61]. The loading and unloading time of 10 bales to and from the trailer would be around 1.5 h. Transport costs of one tractor and trailer would be €1.5/km. So, it is estimated that production costs of 40,000 bales of straw and delivering them to the local depot will cost around €700,000. If autumn is rainy and plastic coating is needed for the majority of bales, the cost will be approximately €800,000. This will be around €50/ton (without plastic coating) and approximately €55–60/ton if plastic is needed. When transporting bales from the depot to the cellulose factory, one HCT truck can take a cargo of 180 m^3^, which is about 70–80 bales. This transport costs about €2/km in both directions. Thus, transporting 40,000 bales will require 500–550 truckloads.

When local pretreatment is used, raw material will drop roughly to 6000–8000 tons depending on the treatment process conditions. The remaining material could be used locally for energy recovery. When the material is processed, the maximum truck cargo load of 48 tons will be a restricting factor. Therefore, 130–160 truckloads between the depot and the cellulose factory will be needed when local pretreatment is used in the depot.

If the pretreatment is used in local depots, around 70% (350–400) fewer truckloads between one local depot and the cellulose factory are needed. For example, if the distance between these two places is around 250 km, local pretreatment will produce €350,000–400,000 savings in transportation costs in one depot. Local pretreatment equipment can be placed in one container, making it very mobile. Though, the production costs will be higher when the production is shared between two locations. However, the cost savings in transportation are remarkable, and when more information about the pretreatment costs is obtained, the economic distance where the pretreatment is justified can be calculated. Furthermore, if the CO_2_ emissions of these two options are compared and valued, the economic distance to apply pretreatment would surely be shorter.

## 5. Conclusions

Competition for wood as a cellulose raw material source is increasing. Straw contains about 30% of cellulose, making it a potential alternative for wood as a raw material source.

The bonding tests in our research indicate that mild pretreatments might be sufficient to enhance the properties of straw (without additional substances) so that it can be used in specific applications, such as insulation and takeaway packaging, which are foreseen to have steady market growth. However, the material preparation requires combining unit processes intelligently to overcome the extensive size distribution of the preprocessed fiber fractions and their relatively poor bonding ability in aqueous environments. Here we proposed using aqueous foam to distribute fibers evenly into a sheet structure and then applying hot pressing to bond the structure together. These operations provided quite strong materials whose physical properties are affected by the pretreatment conditions.

Replacing wood-based cellulose with straw often requires significant changes in how raw material is collected, treated, processed and business is done. In other words, changing raw materials may require changes in existing business models or developing entirely new models. In general, developing a business model requires considering market demand and other key elements, such as raw material supply, storage, logistics, and processing. Furthermore, creating a successful business model that supports a circular economy, such as in the case of straw use, also requires tight collaboration and changes in the entire value chain.

If the pretreatment is used in local depots using mobile container units, considerable savings in transportation costs can be achieved. Furthermore, including CO_2_ emissions in the comparison shortens the economic distance of a possible pretreatment application even further.

## Figures and Tables

**Table 1:** Examples of current applications for cereal straw (barley and wheat).

Fraction	Application	Source
Straw as such	Animal feed	[12]
	Soil amendment, fertilizer, garden mulch, biochar for cultivation and to remove heavy metals	[12,13,14,15]
	Mushroom culture	[12]
	Oil spill sorbent	[16]
	Algae growth control in water systems	[17]
	Energy and Power	[12,18]
	Thermal insulation material for construction and packaging	[19,20]
	Strawboard and MDF board	[13,21,22]
Fibers (cellulose)	Textiles	[23]
	Nonwovens	[24]
	Pulp, paper and board	[8,25]
	Biocomposites	[26]
Monomers and polymers (cellulose, hemicelluloses, lignin, waxes)	Chemicals: e.g., xylose, furfural, acetic acid and other intermediates	[27]
	Bioplastics and adhesives; bio-resins, polyurethane, plasticizers, adhesives (e.g., for construction materials)	[27]
	Films and coatings	[28]
	Waxes: biobased pesticides, polycosanols and sterols as cholesterol-reducing agents	[13]
	Biofuels: Ethanol	[7,27]
	Food and cosmetics ingredients, including waxes	[13]

**Table 2:** Test methods applied for the straw sheets.

Property	Standard
Grammage (g/m^2^)	ISO 5270 (2012), ISO 534 (2011)
Thickness (µm)	
Apparent sheet density (kg/m^3^)	
Bulk (cm^3^/kg)	
Tensile index (Nm/g)	ISO 5270 (2012), EN ISO 1924-2:2008
Strain at break (%)	
Tensile energy absorption index (J/g)	
Tensile stiffness index (kNm/g)	
Wet strength measurements	ISO 3781 (2011)

**Table 3:** Conditions and results in the straw processing experiments.

Biomass NaOH Charge/Temp	Oat 12/80	Barley 12/80	Oat 12/160	Oat 18/160
NaOH charge, % of biomass	12	12	12	18
Liquor-to-straw ratio	6	6	6	6
Temperature, °C	80	80	160	160
Time, min	60	60	60	60
**Liquids:**				
pH	12.6	12.4	10.9	12.5
**Solids:**				
Yield, %	58	58	46	41
Kappa number	48.5 ± 0.3	50.2 ± 0.2	16.6 ± 0.1	16.3 ± 0.0
Brightness, %	12.1 ± 0.2	9.6 ± 0.4	26.5 ± 1.5	26.5 ± 1.6

**Table 4:** Properties of sheets made from unrefined (unref.) and refined straw. For the refined Oat 12/80 trial point, also data with a special enzyme treatment and 30-min hot pressing are included. Wet strength was measured after 1 h of water immersion.

Property	Oat 12/80				Barley 12/80		Oat 12/160		Oat 18/160	
	Unref.	Refined	Enzyme Added	Press 30 min	Unref.	Refined	Unref.	Refined	Unref.	Refined
Grammage, g/m^2^	122	116	113	119	111	113	121	119	100	101
Thickness, µm	208	196	214	177	220	185	191	198	162	159
Density, kg/m^3^	587	592	528	672	505	611	634	601	617	635
DRY										
Tensile index, Nm/g	11.7 ± 2.6	32.5 ± 4.3	13.0 ± 5.2	7.0 ± 2.4	10.9 ± 4.4	26.2 ± 2.5	24.4 ± 4.2	34.7 ± 6.6	21.2 ± 3.5	24.9 ± 4.2
Stretch at break, %	1.0 ± 0.1	1.9 ± 0.3	1.0 ± 0.2	0.7 ± 0.2	0.9 ± 0.3	1.6 ± 0.2	1.9 ± 0.3	2.7 ± 0.5	2.1 ± 0.4	2.5 ± 0.6
TEA * index, J/g	0.08 ± 0.02	0.37 ± 0.10	0.10 ± 0.06	0.04 ± 0.02	0.06 ± 0.05	0.24 ± 0.04	0.29 ± 0.10	0.60 ± 0.22	0.29 ± 0.11	0.42 ± 0.18
Tensile stiffness, Nm/g	490 ± 130	750 ± 70	570 ± 150	420 ± 120	540 ± 140	700 ± 40	590 ± 70	720 ± 80	640 ± 70	690 ± 80
WET										
Solids content, %	38.6	38.5	36.0	44.1	33.4	35.3	36.1	33.4	32.3	35.4
Tensile index, Nm/g	0.5 ± 0.1	1.6 ± 0.4	0.4 ± 0.2	0.9 ± 0.4	0.5 ± 0.1	1.4 ± 0.2	0.9 ± 0.3	1.3 ± 0.3	0.9 ± 0.1	1.2 ± 0.2
Stretch at break, %	1.9 ± 0.4	2.7 ± 0.4	2.1 ± 0.3	2.1 ± 0.4	1.8 ± 0.3	3.3 ± 0.8	2.4 ± 0.4	2.6 ± 0.5	2.6 ± 0.5	2.8 ± 0.7
TEA index, J/g	0.010 ± 0.003	0.03 ± 0.01	0.009 ± 0.004	0.018 ± 0.007	0.008 ± 0.002	0.035 ± 0.009	0.018 ± 0.009	0.03 ± 0.01	0.018 ± 0.004	0.02 ± 0.01
Tensile stiffness, Nm/g	14 ± 5	34 ± 10	13 ± 6	23 ± 14	15 ± 3	24 ± 5	21 ± 7	28 ± 5	22 ± 4	27 ± 3
Top contact angle, °										
0–0.9 s	72 ± 8	80 ± 3	81 ± 4	108 ± 7	66 ± 7	n.m. ***	66 ± 6	n.m.	51 ± 6	n.m.
10–11 s	59 ± 5	60 ± 6	**	97 ± 8	31 ± 6	n.m.	39 ± 12	n.m.	**	n.m.
Bottom contact angle, °										
0–0.9 s	92 ± 6	76 ± 2	67 ± 8	121 ± 6	76 ± 14	n.m.	68 ± 3	n.m.	38 ± 8	n.m.
10–11 s	74 ± 2	63 ± 8	**	118 ± 5	63 ± 15	n.m.	48 ± 7	n.m.	**	n.m.

* Tensile energy absorption. ** Absorption of the droplet is fast. *** Not measured.

## Appendix A

**Table A1:** An overview of potential strengths, weaknesses, opportunities and threats related to residual straw use as a raw material (based on the literature, expert interviews, and the authors’ assessment).

STRENGTH	WEAKNESS
Supports circular bioeconomy improves material efficiency [55] additional biobased raw material source the raw material that can be processed for a variety of end-use applications, industries and products, and can be used as “drop-in” material [27,55] Potentially lower environmental impact [8,10,55] lower CO_2_ emissions compared to wood pulp production lower energy and water use compared to some other cellulose sources, e.g., wood Low impact on land use the use of residual straw (from food production) as an alternative cellulose source does not require changes to land use Supports local economies selling straw offers side incomes for the farmers [56]	Availability and supply security of straw is uncertain the amount of straw to be harvested is hard to estimate due to the changing weather conditions it is estimated that the amount of straw residue is insufficient for industrial scale biorefining in Finland straw availability is highly site-specific and geographically determined (e.g., North America vs. Finland) Infrastructure for the collection and storage of straw is missing [57] the farmers should know in advance if the straw is collected to use the proper adjustments in the combine harvester the farmers don’t necessarily have the machinery or time to collect straw from their fields Challenges in logistics and supply chains [57] relatively short transportation distance to ensure economic feasibility lack of existing supply chains Low technological and business maturity lack of industrial-scale processes or processors in Finland Information missing on cost and revenue logic
OPPORTUNITY	THREAT
Green growth development towards a circular bioeconomy requires significant growth and diversification in sustainable biomass supply, and straw is one option for this possibility to bring more agro-business to the countryside brand values for the companies using straw as an alternative cellulose source Changes in legislation and consumer behavior demand for fossil-free and plastic-free solutions (e.g., in packaging, insulation) is growing impacts of EU policies (e.g., biodiversity, climate and forest policies) on forest-based cellulose fiber supply	Overharvesting harvesting more straw than sustainably available raises concerns regarding, e.g., detrimental effects on the soil humus content [56] competition: lower-value applications (farming, construction, etc.) vs. high-value biorefining (chemicals, textiles, etc.) Insufficient economic incentives the price of straw residue needs to be low enough to allow profitability for straw processors but might not be sufficient to motivate farmers (exchange economy currently in use) Deterioration of raw material quality straw material handling should be performed carefully to avoid deterioration of quality due to, e.g., humidity and mold Impact of climate change on straw supply extreme weather conditions [56]
